# *NQO1* gene rs1800566 variant is not associated with risk for multiple sclerosis

**DOI:** 10.1186/1471-2377-14-87

**Published:** 2014-04-23

**Authors:** José A G Agúndez, Elena García-Martín, Carmen Martínez, Julián Benito-León, Jorge Millán-Pascual, Patricia Calleja, María Díaz-Sánchez, Diana Pisa, Laura Turpín-Fenoll, Hortensia Alonso-Navarro, Lucía Ayuso-Peralta, Dolores Torrecillas, José Francisco Plaza-Nieto, Félix Javier Jiménez-Jiménez

**Affiliations:** 1 Department of Pharmacology, University of Extremadura, Cáceres, Spain; 2 Department of Biochemistry and Molecular Biology, University of Extremadura, Cáceres, Spain; 3 Department of Pharmacology, University of Extremadura, Badajoz, Spain; 4 CIBERNED,Centro de Investigación Biomédica en Red de Enfermedades Neurodegenerativas, Instituto de Salud Carlos III, Madrid, Spain; 5 Service of Neurology, Hospital Universitario Doce de Octubre, Madrid, Spain; 6 Department of Medicine, University Complutense, Madrid, Spain; 7 Section of Neurology, Hospital La Mancha-Centro, Alcázar de San Juan, Ciudad Real, Spain; 8 Centro de Biología Molecular Severo Ochoa (CSIC-UAM), Facultad de Ciencias, Universidad Autónoma, Cantoblanco, Madrid 28049, Spain; 9 Section of Neurology, Hospital Universitario del Sureste, Arganda del Rey, Madrid, Spain; 10 Department of Medicine-Neurology, Hospital “Príncipe de Asturias”, Universidad de Alcalá, Alcalá de Henares, Madrid, Spain

**Keywords:** Multiple sclerosis, Genetics, Genetic polymorphisms, NQO1, Risk factors

## Abstract

**Background:**

A possible role of oxidative stress in the pathogenesis of multiple sclerosis (MS) and in experimental autoimmune encephalomyelitis has been suggested. The detoxification enzyme NAD(P)H dehydrogenase, quinone 1 (NQO1) has been found up-regulated in MS lesions. A previous report described an association between the SNP rs1800566 in the *NQO1* gene and the risk for MS in the Greek population. The aim of this study was to replicate a possible influence of the. SNP rs1800566 in the *NQO1* gene in the risk for MS in the Spanish Caucasian population.

**Methods:**

We analyzed allelic and genotypic frequency of *NQO1* rs1800566 in 290 patients with MS and 310 healthy controls, using *TaqMan* Assays.

**Results:**

*NQO1* rs1800566 allelic and genotypic frequencies did not differ significantly between MS patients and controls, and were unrelated with age of onset of MS, gender, and clinical type of MS.

**Conclusions:**

Our results indicate that NQO1 rs1800566 does not have an effect on MS disease risk.

## Background

Multiple sclerosis (MS) is a chronic inflammatory demyelinating disorder with axonal degeneration of the central nervous system. Although the etiology of MS is not well understood, the interplay of both genetic and environmental factors is the most likely hypothesis [1-5]. With regard to the role of genetic factors, genome-wide association studies (GWAS) have confirmed more than 100 loci with genome-wide significance [6-8]. According to the most recent GWAS, approximately 22% of signals overlapped at least one other autoimmune disease signal [8], However, all loci except HLA showed modest odds-ratio (OR) in the range of 1.1-1.3 [9]. In particular the association between MS and the HLA-DRB1*15:01 haplotype has been shown to be strong.

A number or reports have suggested a possible role of oxidative stress and lipid peroxidation in the inflammatory processes and in the pathogenesis of MS [10-40]. Reactive oxygen species (ROS) generated in excess primarily by macrophages and microglia, have been implicated as mediators of demyelination and axonal damage [10]. The main results of studies on oxidative stress markers in the brain, spinal cord, and CSF, both in MS patients and in experimental autoimmune encephalomyelitis (EAE) are summarized in a table included as an Additional file 1: Table S1.

NAD(P)H dehydrogenase, quinone 1 (NQO1) is a phase II detoxification enzyme that catalyzes the one-electron reduction of endogenous and exogenous quinones, preventing their participation in redox cycling and subsequent generation of reactive oxygen species. This enzyme is encoded by the *NQO1* gene (chromosome 16q22.1, Gene Identity 1728) (link http://www.ncbi.nlm.nih.gov/gene/1728). The enzymatic activity of NQO1 depends fundamentally on a single nucleotide polymorphism (SNP) at the NQO1 locus, rs1800566 (C609T), which produces a proline-to-serine substitution at amino acid 187 (P187S) (link http://www.ncbi.nih.gov/pubmed/9000600). Individuals with rs1800566TT genotype completely lack NQO1 activity, whereas those with rs1800566C/T genotype present approximately threefold decreased enzyme activity [41].

Although *NQO1* polymorphisms are not mentioned among the possible susceptibility genes in GWAS studies, the possible role of oxidative stress in the pathogenesis of MS makes it reasonable to analyse the possible relationship between *NQO1* gene polymorphisms and the risk of MS. Moreover, NQO1 has been found to be markedly up-regulated in active demyelinating MS lesions [13,14] Stavropoulou et al. [42], in a case–control association study involving 231 MS patients and 380 controls, reported an association between the rs1800566CT and TT genotypes and the risk of developing MS, and a higher incidence of rs1800566CT genotype in patients with primary progressive MS. The aim of the present study was to replicate the findings by Stavropoulou et al. [42] in the Spanish population.

## Methods

### Patients and controls

We studied 290 unrelated Caucasian Spanish patients who fulfilled McDonald’s criteria for definite MS [43], with no other previous neurological diseases. MS patients were recruited from the “Multiple Sclerosis Association of Madrid” (n = 165 cases), the Health Areas of the La-Mancha-Centro Hospital (Alcázar de San Juan, Ciudad Real, n = 65 cases), and University Hospitals “Doce de Octubre” (Madrid, n = 30 cases), and “Príncipe de Asturias” (Alcalá de Henares, Madrid, n = 30 cases). Most of these patients had participated in previous studies of genetic association with MS risk [44-48]. The control group was composed of 310 healthy unrelated Caucasian Spanish individuals (students or professors from the University of Extremadura) gender and age-matched with the patients. Ethnicity and geographical origin for cases and controls was self-reported. All the participants were included in the study after giving written informed consent. Table 1 summarizes the characteristics of the individuals included in the study. The protocol was approved by the Ethics Committees of the University Hospitals “Príncipe de Asturias” and “Infanta Cristina” (Badajoz). The study was conducted according to the principles expressed in the declaration of Helsinki.

**Table 1:** Characteristics of the individuals included in the study

	**Overall MS patients**	**RRMS**	**SPMS**	**PPMS**	**Control individuals**
Gender (females/males)	200/90	111/44	59/33	30/13	213/97
Age (mean ± SD)	43.8 ± 11.3	40.0 ± 10.5	47.2 ± 9.9	54.4 ± 10.2	43.7 ± 12.2
Age at onset (mean ± SD)	32.6 ± 10.6	29.5 ± 8.5	34.9 ± 11.8	43.4 ± 11.6	-
Disease duration (mean ± SD)	11.2 ± 7.9	10.5 ± 8.2	12.3 ± 9.3	11.0 ± 7.7	-
Expanded disability status scale (EDSS) (median, Interquartile range)	3.0, 4.50	2.5, 2.00	6.5, 0.90	7.5, 0.50	-
Progression index (EDSS/MS duration)	0.5 ± 0.4	0.3 ± 0.2	0.6 ± 0.5	0.7 ± 0.4	-

RRMS Relapsing remitting Multiple Sclerosis. SPMS Secondary Progressive Multiple Sclerosis. PPMS Primary Progressive Multiple Sclerosis.

### Genotyping of rs1800566 polymorphism

Genotyping for *rs1800566* was performed in genomic DNA obtained from venous blood samples of participants using *TaqMan* Assays (C___2091255_30, Life Technologies, Alcobendas, Madrid, Spain). Detection was carried out by qPCR in an Eppendorf realplex thermocycler. The amplification conditions were as follows: after a denaturation time of 10 min at 96°C, 45 cycles of 92°C 15 sec 60°C 90 sec were carried out and fluorescence was measured at the end of each cycle and at endpoint. All samples were determined in triplicate and genotypes were assigned both by gene identification software (RealPlex 2.0, Eppendorf, Madrid, Spain) and by analysis of the reference cycle number for each fluorescence curve, calculated by the use of the CalQPlex algorithm (Eppendorf, Madrid, Spain).

### Statistical analysis

Hardy-Weinberg equilibrium (HWE) both in MS patient and control groups was analyzed by the DeFinetti software (http://ihg.gsf.de/cgi-bin/hw/hwa1.pl). Allele and genotype frequency analysis was performed with SPSS ver. 17.

For categorical variables the intergroup comparison values were calculated using the chi-square or Fisher’s exact tests when appropriate. For continuous variables, the Kolmogorov-Smirnoff test was used to analyze normality in the distribution. Then, the Student two sample *t* test was used for variables that followed a normal distribution (age and age at onset); the Mann–Whitney test was used for the duration of disease and the severity scores expanded disability status scale and progression index, because no normal distribution was observed for this parameter. The association between the genotypes and the risk of developing MS was assessed by two-way contingency table analysis (http://statpages.org/ctab2x2.html). Statistical power was calculated for the sample size of this study (this was determined from allele frequencies with a genetic model analyzing the frequency for carriers of the disease gene with Odds ratio (OR) = 1.5, p = 0.05 [49]). Two-tailed and one-tailed associations of the risk with the variant allele for ORs of 1.5; 1.4; 1.3; 1.2; and 1.1; were, respectively: 84.6% and 90.9%; 68.9% and 79.0%; 47.4% and 59.8%; 25.6% and 36.6%; and 9.9% and 16.56%.

## Results

The frequencies of *NQO1* rs1800566 genotypes and allelic variants in patients diagnosed with MS did not differ from those of controls (Table 2). The genotype and allele frequencies in MS patients and healthy subjects were in Hardy-Weinberg’s equilibrium. Mean age at onset of MS did not differ significantly between patients carrying *NQO1* rs1800566 C/C (mean ± SD = 32.1 ± 10.2 years, reference), C/T (mean ± SD = 33.5 ± 11.6 years, p = 0.220) and T/T (mean ± SD = 32.4 ± 12.0 years, p = 0.270). Association between the rs1800566 variant and MS risk was not observed when analyzing gender separately (Table 2). The distribution of the *NQO1* rs1800566 genotype and allelic frequencies did not differ between each MS clinical evolutive type and controls (Table 3) or in the severity scores: Expanded Disability Status Score or EDSS (p = 0.508 and p = 0.370 for heterozygous and homozygous carriers of the minor allele, respectively, as compared with homozygous carriers of the major allele) or progression index (p = 0.872 and p = 0.673 for heterozygous and homozygous carriers of the minor allele, respectively, as compared with homozygous carriers of the major allele).

**Table 2:** ***NQO1 rs1800566 *** **genotype and allelic variants of patients with multiple sclerosis (MS) and healthy volunteers**

	**MS PATIENTS N (%, 95% C.I.)**	**CONTROLS N (%, 95% C.I.)**	**Intergroup comparison values OR (%, 95% C.I.) P**	**MS WOMEN N (%, 95% C.I.)**	**CONTROL WOMEN N (%, 95% C.I.)**	**Intergroup comparison values OR (%, 95% C.I.) P**	**MS MEN N (%, 95% C.I.)**	**CONTROL MEN (N (%, 95% C.I.)**	**Intergroup comparison values OR (95% CI) P**
rs1800566 GENOTYPE C/C	178 (61.4, 55.8-67.0)	195 (62.9, 57.5-68.3)	Reference	120 (60.0, 53.2-66.8)	134 (62.9, 56.4-69.4)	Reference	58 (64.4, 54.6-74.3)	61 (62.9, 53.3-72.5)	Reference
C/T	99 (34.1, 28.7-39.6)	104 (33.5, 28.3-38.8)	1.43 (0.74-1.47), 0.810	72 (36.0, 29.3-42.7)	72 (33.8, 27.5-40.2)	1.12 (0.74-1.68), 0.597	27 (30.0, 20.5-39.5)	32 (33.0, 23.6-42.3)	0.88 (0.48-1.66), 0.708
T/T	13 (4.5, 2.1-6.9)	11 (3.5, 1.5-5.6)	1.30 (0.57-2.96), 0.540	8 (4.0, 1.3-6.7)	7 (3.3, 0.9-5.7)	1.28 (0.45-3.63), 0.646	5 (5.6, 0.8-10.3)	4 (4.1, 0.2-8.1)	1.32 (0.34-5.14), 0.693
Total	290	310		200	213		90	97	
Allele C	455 (78.4, 75.1-81.8)	494 (79.7, 76.5-82.8)	Reference	312 (78.0, 73.9-82.1)	340 (79.8, 76.0-83.6)	Reference	143 (79.4, 73.5-85.3)	154 (79.4, 73.7-85.1)	Reference
Allele T	125 (21.6, 18.2-24.9)	126 (20.3, 17.2-23.5)	1.08 (0.82-1.42), 0.601	88 (22.0, 17.9-26.1)	86 (20.2, 16.4-24.0)	1.12 (0.80-1.56), 0.523	37 (20.6, 14.7-26.5)	40 (20.6, 14.9-26.3)	1.00 (0.60-1.65), 0.988
Total alleles	580	620		400	426		180	194	

Major alleles and genotypes were assumed as reference values. P values correspond to logistic regression analyses.

**Table 3:** ***NQO1 rs1800566 *** **genotype and allelic variants in patients with multiple sclerosis (MS), and relation with the clinical evolutive type of MS**

	**BOUT ONSET MS (RELAPSING REMITTING PLUS SECONDARY PROGRESSIVE MS) N**	**Intergroup comparison values OR (95% CI), P**	**PRIMARY PROGRESSIVE MS N (%, 95% C.I.)**	**Intergroup comparison values OR (95% CI), P**	**CONTROLS N (%, 95% C.I.)**
rs1800566 GENOTYPE C/C	153 (61.9; 55.9-68.0)	Reference	25 (58.1, 43.4-72.9)	Reference	195 (62.9, 57.5-68.3)
C/T	85 (34.4; 28.5-40.3)	1.04 (0.72-1.51); 0.822	14 (32.6, 18.6-46.6)	1.05 (0.52-2.11), 0.891	104 (33.5, 28.3-38.8)
T/T	9 (3.6; 1.3-6.0)	1.04 (0.39-2.79); 0.928	4 (9.3, 0.6-18.0)	2.84 (0.84-9.59), 0.081	11 (3.5, 1.5-5.6)
Total	247		43		310
Allele C	391 (79.1; 75.6-82.7)	Reference	64 (74.4, 65.2-83.6)	Reference	494 (79.7, 76.5-82.8)
Allele T	103 (20.9; 17.3-24.4)	1.03 (0.76-1.40); 0.829	22 (25.6, 16.4-34.8)	1.35 (0.80-2.27), 0.262	126 (20.3, 17.2-23.5)
Total alleles	494		86		620

All subgroups were compared with control subjects. Major alleles and genotypes were assumed as reference values. P values correspond to logistic regression analyses.

## Discussion

In contrast with the findings in the study by Stavropoulou et al. [42], we did not find significant differences either in the frequencies of rs1800566 genotypes, or in the frequencies of the allelic variants of this polymorphism in patients with MS when compared with healthy controls. In addition, rs1800566 polymorphism was neither associated with age at onset of MS, nor with clinical type of MS. Patient sample size and statistical power are higher in our study than in the study by Stavropoulou et al. [42].

A possible reason for the discrepancies between the study by Stavropoulou et al. [42] and the present one is the fact that the genotype distribution in the control group of the Stavropoulou et al. [42] study was in Hardy-Weinberg’s disequilibrium (Pearson’s p = 0.0086). The Hardy-Weinberg’s disequilibrium was attributable to the female control group of the study by Stavropoulou et al. [42]. Another putative difference is that in the study by Stavropoulou et al. [42] the genotyping analysis was carried out by using qPCR melting curves, whereas in our study TaqMan genotyping was used.

Several SNPs have been described within the *NQO1* gene. It should be noted that most association studies on *NQO1* focusing on the SNP analyzed in this study show a relatively high minor allele frequency (about 20% for Caucasian populations according to 1000 genomes). The rs1800566 analyzed in this study is common in Caucasian individuals; in fact it occurs in all human populations, and is classified as a pathogenic allele related with altered susceptibility to benzene toxicity and response to chemotherapy (see http://browser.1000genomes.org/Homo_sapiens/Variation/Phenotype?db=core;r=16:69744645–69745645;v=rs1800566;vdb=variation;vf=1366399).

The present study has some limitations. First, the size of the analyzed cohorts may not be sufficient for strict conclusions about the role of *NQO1*in MS (though adequate to detect an OR as small as 1.5, a more modest association would not be detected). Secondly, because the cohort study included MS patients with different degrees of severity, it is not adequate for the investigation of the influence of *NQO1* genotypes on disability or severity of MS. The ideal study for this purpose should be prospective, including the genotyping of patients with a recent diagnosis of MS and a re-examination of the same patient cohort after similar long-term follow-up periods to establish evolutive type).

## Conclusions

Taking in account the limitations of the present study, the results suggest that, in contrast with the Stavropoulou et al. [42] report, *NQO1* rs1800566 genotypes and allelic variants are not associated with the risk for MS in Caucasian Spanish people. The fact that this particular SNP showed lack of association with MS risk in the present study does not exclude the possibility that other SNPs in the *NQO1* gene could be associated with a modification in the risk of this disease.

## Competing interest

The authors declare that they have no competing interests.

## Authors’ contributions

JAGA participated in the conception and design of the study, acquisition of data, analysis and interpretation of data, drafting of the manuscript, critical revision of the manuscript, administrative, technical, and material support, supervision, and obtaining funding. EGM participated in the conception and design of the study, acquisition of data, analysis and interpretation of data, drafting of the manuscript, critical revision of the manuscript, administrative, technical, and material support, supervision, and obtaining funding. CM participated in acquisition of data, analysis and interpretation of data, critical revision of the manuscript, administrative, technical, and material support. JBL participated in acquisition of data, and critical revision of the manuscript. JMP participated in acquisition of data, and critical revision of the manuscript. PC participated in acquisition of data, and critical revision of the manuscript. MDS participated in acquisition of data, and critical revision of the manuscript. DP participated in acquisition of data, and critical revision of the manuscript. LTF participated in acquisition of data, and critical revision of the manuscript. HAN participated in acquisition of data, analysis and interpretation of data, critical revision of the manuscript, administrative, technical, and material support. LAP participated in acquisition of data, and critical revision of the manuscript. DT participated in acquisition of data, and critical revision of the manuscript. JFPN participated in acquisition of data, and critical revision of the manuscript. FJJJ participated in the conception and design of the study, acquisition of data, analysis and interpretation of data, drafting of the manuscript, critical revision of the manuscript, administrative, technical, and material support, and supervision. All authors read and approved the final manuscript

## Pre-publication history

The pre-publication history for this paper can be accessed here:

http://www.biomedcentral.com/1471-2377/14/87/prepub

## Supplementary Material

Additional file 1: Table S1 Results of studies of oxidative stress markers in the brain, spinal cord, and CSF of MS patients and in experimental autoimmune encephalomyelitis (EAE). Click here for file
