# Supplementary material for: NQO1 gene rs1800566 variant is not associated with risk for multiple sclerosis
Source: BMC Neurol. 2014 Apr 23;14:87. doi: 10.1186/1471-2377-14-87 (PMC4022329; doi:10.1186/1471-2377-14-87)
Supplement: Additional file 1: Table S1 — Results of studies of oxidative stress markers in the brain, spinal cord, and CSF of MS patients and in experimental autoimmune encephalomyelitis (EAE). [file 1471-2377-14-87-S1.doc]

**Additional file 1: Table S1. Results of studies of oxidative stress markers in the brain, spinal cord, and CSF of MS patients and in experimental autoimmune encephalomyelitis (EAE)**

| **BRAIN AND SPINAL CORD TISSUE OF MS PATIENTS** |
| --- |
| Presence of oxidized LDL and lipid peroxidative end-products (malondialdehyde, 4-hydroxynonenal, oxidized phospholipids epytopes) in early and actively demyelinating plaques [11, 12]. |
| Increased oxidized DNA in active MS plaques [12] |
| Presence of extensive oxidative damage to proteins, lipids, and nucleotides, together with a marked up-regulation the antioxidant enzymes superoxide-dismutases 1 and 2, catalase, heme oxygenase 1, and NAD(P)-quinone oxidreductase 1 (NQO1), in active demyelinating MS lesions [13, 14] |
| Increased amounts of protein carbonyls in the brain white and gray matter in MS patients [15]. |
| Decreased activities of 3 peptidases of the 20S proteasome both in white and gray matter, suggesting failure of the degradation systems that lead to the build-up of carbonylated proteins [16] |
| Increased uric acid and decreased glutathione in plaques, decreased alpha-tocopherol levels in plaques, and increased alpha-tocopherol levels in distant white matter, and normal ascorbic acid, cysteine, tyrosine, and tryptophan levels, both in plaques and in distant white matter [17] |
| Up-regulation of the transcription factor NF-E2-related factor (Nrf2, regulator of endogenous antioxidant enzymes) in macrophages and astrocytes in active lesions, and increased expression of DJ1 protein in astrocytes of active and chronic lesions [18] |
| Increased PINK1 (mitochondrial kinase) immunostaining in astrocytes of active demyelinating lesions, which is decreased in chronic lesions [19] |
| Increased levels of CHOP and normal levels of BIP (endoplasmic reticulum stress molecules) in gray matter demyelinating lesions [20] |
| Up-regulation of the NADPH1 expression in active and slowly expanding MS lesions[21] |
| Decreased GSH levels in the brain of secondary progressive MS patients, especially in the frontal region, by ^1^H-MR chemical swift imaging [22] |
| **BRAIN AND SPINAL CORD TISSUE OF EAE MODELS** |
| Increased amounts of protein carbonyls in white matter astrocytes and to a lesser extent in migroglia/macrophages in both acute and chronic phases in the brain [23], and in the spinal cord (associated to protein aggregation and apoptosis) of experimental EAE models [24] |
| Increased immunoreactivity of nitrotyrosine (an indicator of peroxynitrite formation) in the spinal cord white matter in EAE, which correlated with the loss of mature olygodentrocytes [25] |
| **CEREBROSPINAL FLUID OF MS PATIENTS** |
| Increased levels of lipid peroxidation markers such as pentane and ethane [26], malonil-dialdehyde (MDA) [27, 28], thiobarbituric acid reactive substances (TBARS) [29], isoprostanes [30-32], hydroxynonenal [33], and 8-isoprostaglandin F2alpha [29, 34] |
| Normal levels of advanced glycoxidation end products levels [35]. |
| Increased levels of protein carbonyls [33] and advanced oxidation protein products [36] and decreased levels of total thiol groups [36]. |
| Increased activity of glutathione-reductase and decreased activity of glutathione-peroxidase [27]. |
| Normal alpha-tocopherol levels [37]. |
| Nitrate levels normal [38] or increased [29], and nitrotyrosine levels increased [29] |
| Uric acid levels increased [39] or normal [40]. Normal levels of the uric acid oxidation product allantoine [40], and increased levels of the uric acid precursors hypoxanthine and xanthine [39]. |
| Increased levels of heat shock proteins (Hsp) 72 and 70, and hem-oxygenase 1, and increased expression of thioredoxin (Trx) and sirtuin protein systems and decreased expression of TrxR [33]. |
